# Up to seven-fold inter-hospital differences in obstetric anal sphincter injury rates- A birth register-based study in Finland

**DOI:** 10.1186/1756-0500-3-345

**Published:** 2010-12-23

**Authors:** Sari Räisänen, Katri Vehviläinen-Julkunen, Mika Gissler, Seppo Heinonen

**Affiliations:** 1 Department of Nursing Science, University of Eastern Finland, P.O. Box 1627, 70211 Kuopio, Finland; 2 Kuopio University Hospital, P.O. Box 1777, 70211 Kuopio, Finland; 3 National Institute for Health and Welfare (THL), P.O. Box 30, Lintulahdenkuja 4, Finland; 4 Nordic School of Public Health, P.O.Box 12133/Nya Varvet SE-402 42 Göteborg, Sweden; 5 University of Eastern Finland, P.O. Box 1627, 70211 Kuopio, Finland

## Abstract

**Background:**

The occurrence of obstetric anal sphincter injuries (OASIS) - which may have serious, long-term effects on affected women, including faecal incontinence, despite primary repair - varies widely between countries and have been chosen one of the indicators for patient safety in Organisation for Economic Cooperation and Development (OECD) countries and in Nordic countries.

**Findings:**

The aim of the study was to assess risks of OASIS among five university teaching hospitals and 14 non-university central hospitals with more than 1,000 deliveries annually during 1997-2007 in Finland. Women with singleton vaginal deliveries divided into two populations consisting of all 168,637 women from five university hospitals and all 255,660 women from non-university hospitals, respectively, derived from population-based register. Primiparous and multiparous women with OASIS (n = 2,448) were compared in terms of possible risk factors to primiparous and multiparous women without OASIS, respectively, using stepwise logistic regression analysis. The occurrences of OASIS varied from 0.7% to 2.1% in primiparous and from 0.1% to 0.3% in multiparous women among the university hospitals. Three-fold inter-hospital differences in OASIS rates did not significantly change after adjustment for patient mix or the use of interventions. In non-university hospitals OASIS rates varied from 0.2% to 1.4% in primiparous and from 0.02% to 0.4% in multiparous women, and the results remained virtually unchanged after adjustment for known risks.

**Conclusions:**

Up to 3.2-fold inter-hospital differences in OASIS risk demonstrates significant differences in the quality of Finnish obstetric care.

## Findings

Birth injuries have been chosen one of the 21 indicators for patient safety in Organisation for Economic Cooperation and Development (OECD) countries [1], and one of 36 quality indicators in Nordic countries [2]. Obstetric anal sphincter injury (OASIS) is a serious complication of delivery, which frequently results in faecal incontinence despite primary repair and has serious implications for women's health [3-5]. The occurrence of OASIS varies widely between countries, and might be consequently preventable. For instance, in Finland and Sweden rates of 1% and 4% have been reported [6] respectively. To date, studies on the relationship between OASIS rates and standards of obstetric care have focused primarily on hospital-based data that have recognized vacuum assistance [7,8], forceps delivery [9], midline episiotomy [10], primiparity [9], occiput posterior presentation[7], and high birth weight [9] as risk factors of OASIS.

We have previously reported similar findings with regard to the OASIS risk profile in national-based register data with the exception that lateral episiotomy [11], which is exclusively used in Finland, was associated with decreased risk of OASIS among the primiparous but not the multiparous women [12]. Further, we found a disturbing threefold increase in the risks over an 11-year period from 1997 to 2007. The reason for the increase in OASIS rates has remained mainly obscure since it cannot be explained by changes in the child-bearing population. However, increased use of vacuum assistance explained 9% of it [13]. The aim of the present study was to investigate whether treatment differences by delivery hospitals, volume or teaching status, or the patient mix, have an impact on differences in OASIS rates.

## Methods

This was a retrospective population-based register study, using data obtained for the period 1997 to 2007 from the Medical Birth Register (MBR), which compiles information from clinical records of all of the obstetric care units in Finland and is currently maintained by the National Institute for Health and Welfare (THL). THL, the current register keeper, gave the required authorization for the use of sensitive health register data in scientific research, as required by national data protection legislation. Only anonymised data were used and thus no informed consent of the registered persons was needed.

The MBR includes information on maternal and neonatal birth characteristics and perinatal outcomes (live-born or stillborn infants born after the 22nd gestational week or weighing 500 g or more). Information on OASIS has been collected in the MBR since 2004. For the years 1997-2003, the information was taken from the Hospital Discharge Register with ICD-10 codes O70.2 (3^rd ^degree) and O70.3 (4^th ^degree). The two data sources were linked together with mothers' unique personal identification numbers. The degree of OASIS was classified according to standard definitions: a third degree rupture involves the external anal sphincter and a fourth degree rupture affects both the anal sphincter and anorectal mucosa [14]. The perineum was checked by midwives who asked obstetricians to review the assessment if necessary.

To exclude the possibility that data from small hospitals may have biased the results, we examined data for two populations of women with singleton vaginal deliveries (including all presentations and assisted deliveries) from 19 Finnish hospitals with more than 1,000 deliveries annually during the eleven-year study period (1997-2007), from the total population of women with such deliveries during the period from all 33 Finnish hospitals (mean 2,540, range 133-5,582 in 2007). The hospitals were divided into two groups in terms of their teaching status. Population 1 consisted of 168,637 women (73,813 primiparous and 94,824 multiparous) delivered in five university teaching hospitals (A-E). Population 2 consisted of 255,660 women (109,596 primiparous and 146,064 multiparous) delivered in non-university central (geographic region) hospitals (F-S) divided into three groups by OASIS rate over the 11-year study period. Further, the non-university central hospitals were divided into three groups based on OASIS rates (low, medium or high) over the years 1997-2007. The latter classification was based on an arbitrary, convenient stratification into three groups with approximately equal sizes, with OASIS rates ranging from 0.2% to 1.4% in primiparous and from 0.02% to 0.4% in multiparous women. These populations were analyzed separately because numbers of deliveries in the five university hospitals were among the highest in Finland (range 2,301-4,851 in 2007) and the most complicated deliveries were managed in these units.

The Chi Square test was used to assess the differences in categorical variables between the hospitals. The differences between continuous variables that were not normally distributed were analyzed by using Kruskall-Wallis tests. Differences were deemed to be significant if *p *< 0.05. Multivariate analyses of significant and selected clinically important variables were taken into account in the logistic regression analyses, performed in a stepwise manner, in order to model the risks of OASIS. In all of the analyses, data on third and fourth degree obstetric anal sphincter ruptures were pooled. The data were analyzed using SPSS for Windows 16.0.

In Finland, the active second stage of birth is defined an active phase of bearing down until the delivery of the infant.

## Results

In the five university hospitals, occurrences of OASIS in primiparous women varied from 0.7% to 2.1% (p ≤ 0.001) and from 0.1% to 0.3% (p ≤ 0.001) in multiparous women (Table 1). There were significant differences in use of obstetric interventions between the hospitals, for example in uses of episiotomy and vacuum assistance. Further, the mean duration of active second stage of birth in primiparous women varied between 21-92 minutes (pooled 45 min). In non-university hospitals, the occurrence of OASIS varied from 0.2% to 1.4% (p ≤ 0.001) in primiparous women and from 0.02% to 0.4% (p ≤ 0.001) in multiparous women (Table 2). As shown in Table 2 there were significant differences in the use of most obstetric interventions between non-university hospitals.

**Table 1:** Characteristics and delivery interventions (%) among the primiparous (n = 73,813) and multiparous women (n = 94,824) with singleton vaginal delivery in University Hospitals between 1997 and 2007 in Finland (Chi Square test/Kruskall Wallis test).

CHARACTERISTICS/INTERVENTIONS	Hospital A	Hospital B	Hospital C	Hospital D	Hospital E	p value	Pooled
**PRIMIPAROUS**, **n = 73, 813**	19, 185	12, 486	17, 749	15, 974	8, 419		

Obstetric anal sphincter injuries (OASIS), n (%)	147 (0.8)	127 (1.0)	193 (1.1)	343 (2.1)	56 (0.7)	≤0.001	1.2

Birth weight >4,000 g	13.2	12.6	13.0	13.4	11.7	0.002	12.9

Mean (± SD) length of active second stage of birth (min)	34 ± 31	32 ± 28	92 ± 76	74 ± 61	21 ± 18	≤0.001	45 ± 48

Episiotomy	75.5	64.2	59.2	51.1	49.4	≤0.001	61.4

Vacuum assistance	15.3	15.8	15.9	15.4	10.6	≤0.001	15.0

Amniotomy	49.8	55.2	48.8	31.2	42.4	≤0.001	45.6

Augmentation with oxytocin	75.5	65.2	49.7	53.8	37.7	≤0.001	58.6

Epidural analgesia	62.7	65.7	64.3	78.6	73.7	≤0.001	68.3

Paracervical block	29	24.1	1.0	6.3	10.7	≤0.001	14.4

*Caesarean section	17.7	21.4	31.4	19.0	23.0	≤0.001	22.9

**MULTIPAROUS**, **n = 94, 824**	23, 746	21, 291	20, 628	18, 032	11, 127		

OASIS, n (%)	33 (0.1)	30 (0.1)	52 (0.3)	61 (0.3)	13 (0.1)	≤0.001	0.2

Birth weight >4000 g	24.3	22.3	21.7	24.0	22.2	≤0.001	23.0

Mean (± SD)length of active second stage of birth (min)	11 ± 13	9 ± 12	36 ± 44	21 ± 28	11 ± 11	≤0.001	13 ± 20

Episiotomy	15.2	8.6	20.4	6.5	10.3	≤0.001	12.6

Vacuum assistance	2.5	2.0	2.6	2.1	1.9	≤0.001	2.3

Amniotomy	53.6	60.0	52.3	34.9	43.9	≤0.001	50.1

Augmentation with oxytocin	37.6	35.1	33.8	23.3	20.5	≤0.001	31.5

Epidural analgesia	19.6	14.9	47.5	34.1	26.8	≤0.001	28.2

Paracervical block	44.8	41.6	2.8	25.8	26.2	≤0.001	29.2

*Caesarean section	8.0	9.5	15.7	8.8	12.1	≤0.001	10.7

*Cesarean section rates of each hospital are also given during the same period of time

**Table 2:** Characteristics and delivery interventions (%) in non-university hospitals (F-S) (included if ≥1, 000 deliveries/year) with different obstetric anal sphincter injuries (OASIS) rates in primiparous (n = 109, 596) and multiparous women (n = 146, 064) with singleton vaginal delivery between 1997 and 2007 in Finland (Chi Square test/Kruskall Wallis test).

CHARACTERISTICS/INTERVENTIONS	Low: Hospitals with ≤0.5% OASIS rate	Medium: Hospitals with 0.6-1% OASIS rate	High: Hospitals with 1.1-1.4% OASIS rate	p value	Pooled
**PRIMIPAROUS**, **n = 109, 596 (%)**	15, 529 (14.2)	24, 315 (22.2)	69, 752 (63.6)		

Hospitals, n	3 (G, L, P)	4 (I, M, N, R)	7 (F, H, J, K, O, Q, S)		

OASIS, n = 1,137	61 (0.4)	185 (0.8)	891 (1.3)	≤0.001	1.0

Birth weight >4,000 g	13.0	11.6	12.8	≤0.001	12.5

Mean (± SD) of active second stage of birth (min)	40 ± 36	28 ± 29	45 ± 43	≤0.001	39 ± 39

Episiotomy	75.0	75.7	60.7	≤0.001	66.1

Vacuum assistance	12.9	16.1	14.8	≤0.001	14.8

Amniotomy	46.5	52.1	50.0	≤0.001	50.0

Augmentation with oxytocin	66.7	72.6	66.3	≤0.001	67.7

Epidural analgesia	57.4	61.1	55.8	≤0.001	57.2

Paracervical block	15.6	18.1	11.8	≤0.001	13.7

*Caesarean section	21.9	22.4	22.2	0.47	22.2

**CHARACTERISTICS/INTERVENTIONS**	**Low: Hospitals with <0.1%** **OASIS rate**	**Medium: Hospitals** **with 0.1-0.2%** **OASIS rate**	**High**: **Hospitals** **with 0.3-0.4%** **OASIS rate**	**p value**	**Pooled**

**MULTIPAROUS**, **n = 146, 064 (%)**	12, 128 (8.3)	98, 793 (67.6)	35, 143 (24.1)		

Hospitals, n	2 (G, L)	9(F, H, I, K, M, N, O, P, R)	3 (J, Q, S)		

OASIS, n = 256	4 (0.03)	151 (0.15)	101 (0.29)	≤0.001	0.18

Birth weight >4,000 g	21.8	22.6	23.8	≤0.001	22.8

Mean (± SD) of active second stage of birth (min)	13 ± 18	13 ± 19	12 ± 15	≤0.001	13 ± 18

Episiotomy	21.1	17.4	16.5	≤0.001	17.5

Vacuum assistance	1.9	2.1	2.0	0.17	2.1

Amniotomy	45.8	54.5	56.1	≤0.001	54.1

Augmentation with oxytocin	28.4	39.4	37.0	≤0.001	37.9

Epidural analgesia	11.5	16.7	16.6	≤0.001	16.3

Paracervical block	24.8	22.5	16.5	≤0.001	21.3

*Caesarean section	10.1	10.0	8.6	≤0.001	9.7

*Caesarean section rates of each hospital are also given during the same period of time

Table 3 presents risk-adjusted factors for OASIS among primiparous and multiparous women delivered at each of the five university hospitals. The results show 3.2-fold and 3-fold differences in the risk of having an OASIS for primiparous and multiparous women, respectively, depending on the university hospital where the delivery occurred. The largest risk factors of OASIS included birth weight over 4,000 grams, vacuum assistance, augmentation with oxytocin, and episiotomy for multiparous women. However, it is of note that risks of OASIS were lowest in university teaching hospital with the lowest (E) and the highest (A) use of episiotomy among both groups of women.

**Table 3:** Adjusted Odds Ratios (OR) of obstetric anal sphincter injuries (OASIS) (n = 866/n = 189) among the primiparous (n = 73,813) and the multiparous women (n = 94,824) with vaginal delivery in University Hospitals between 1997 and 2007 in Finland.

CHARACTERISTICS/INTERVENTIONS	Adjusted OR	95% CI	p value
**PRIMIPAROUS**, n = 73,813			

Mode of delivery			
Vaginal spontaneous	1		
Vacuum assistance	2.65	2.29-3.06	≤0.001

Epidural analgesia	0.73	0.62-0.86	≤0.001

Augmentation with oxytocin	1.42	1.2-1.68	≤0.001

Maternal age (year)			
≤19	1		
20-29	1.70	1.11-2.62	0.02
30-39	2.09	1.35-3.24	0.001
≥40	1.65	0.85-3.22	0.14

Birth weight (g)			
≤2,999	1		
3,000-3,499	1.4	1.44-2.62	≤0.001
3,500-3,999	3.01	2.25-4.03	≤0.001
≥4,000	4.97	3.67-6.74	≤0.001

Hospital A	1		
Hospital B	1.37	1.08-1.74	0.01
Hospital C	1.52	1.21-1.91	≤0.001
Hospital D	3.22	2.64-3.93	≤0.001
Hospital E	1.11	0.81-1.52	0.53

**MULTIPAROUS**, n = 94,824			

Mode of delivery			
Vaginal spontaneous	1		
Vacuum assistance	2.23	1.32-3.76	0.003

Episiotomy	2.95	2.07-4.18	≤0.001

Augmentation with oxytocin	1.36	1.00-1.84	0.05

Birth weight (g)			
≤2,999	1		
3,000-3,499	0.91	0.40-2.07	0.83
3,500-3,999	2.48	1.20-5.15	0.014
≥4,000	3.73	1.80-7.77	≤0.001

Hospital A	1		
Hospital B	1.14	0.69-1.87	0.61
Hospital C	1.91	1.23-2.97	0.004
Hospital D	3.03	1.97-4.67	≤0.001
Hospital E	1.0	0.53-1.92	0.99

Odds ratios (OR) adjusted for mode of delivery, hospitals, induction, oxytocin, episiotomy, occiput posterior presentation (OP), epidural analgesia, spinal analgesia, use of nitrous oxide gas, paracervical block, maternal age, body mass index [BMI = body weight in kilograms/height in meters squared], length of active second stage of birth, birth weight, and head circumference. OP, spinal analgesia, BMI, length of active second stage of birth, and head circumference were adjusted for the years 2004-2007. All continuous variables (maternal age, BMI, birth weight, head circumference, and length of active 2^nd ^stage of birth) were classified as categorical variables.

Table 4 presents risk-adjusted factors for OASIS among women delivered at the 14 non-university central hospitals classified by OASIS rate. There were up to 3-fold differences in risks of OASIS for primiparous and 8.2-fold for multiparous among these hospitals. The risk profile of OASIS was fairly consistent with university hospitals. It is of note that the hospitals that had high rates of OASIS among primiparous women also tended to have high rates among multiparous women.

**Table 4:** Adjusted Odds Ratios (OR) of obstetric anal sphincter injuries (OASIS) (n = 1,137/n = 256) among primiparous (n = 109,596) and multiparous women with vaginal delivery (n = 146,064) in Non-University central Hospitals divided into three groups based on OASR rates (Low, Medium, High) between 1997 and 2007 in Finland.

CHARACTERISTICS/INTERVENTIONS	Adjusted OR	95% CI	p value
**PRIMIPAROUS**, n = 109,596			

Mode of delivery			
Vaginal spontaneous	1		
Forceps	24.40	6.90-86.29	≤0.001
Vacuum assistance	4.76	3.70-6.14	≤0.001

Episiotomy and vaginal spontaneous	1		
Episiotomy and forceps	0.18	0.04-0.75	0.018
Episiotomy and vacuum assistance	0.50	0.38-0.65	≤0.001

Epidural analgesia	0.79	0.70-0.89	≤0.001

Maternal age (year)			
≤19	1		
20-29	1.74	1.22-2.49	0.002
30-39	2.23	1.56-3.21	≤0.001
≥40	1.56	0.78-3.12	0.21

Birth weight (g)			
≤2,999	1		
3,000-3,499	1.91	1.47-2.47	≤0.001
3,500-3,999	2.74	2.12-3.53	≤0.001
≥4,000	4.27	3.27-5.59	≤0.001

Hospitals OASIS rate			
Low: ≤0.5%	1		
Medium: 0.6-1%	1.84	1.38-2.47	≤0.001
High: 1.1-1.4%	2.97	2.29-3.85	≤0.001

**MULTIPAROUS**, n = 146, 064			

Mode of delivery			
Vaginal spontaneous	1		
Vacuum assistance	8.33	4.78-14.54	≤0.001

Episiotomy and vaginal spontaneous	1		
Episiotomy and vacuum assistance	0.41	0.20-0.83	0.01

Episiotomy	2.82	2.12-3.76	≤0.001

Epidural analgesia	1.38	1.03-1.84	0.03

Birth weight (g)			
≤2,999	1		
3,000-3,499	2.03	0.80-5.21	0.14
3,500-3,999	3.92	1.61-9.76	0.003
≥4,000	7.56	3.15-19.0	≤0.001

Hospitals OASIS rate			
Low: ≤0.1%	1		
Medium: 0.1-0.2%	4.37	1.62-11.81	0.004
High: 0.3-0.4%	8.24	3.03-22.41	≤0.001

Odds ratios (OR) adjusted for mode of delivery, hospitals, induction, oxytocin, episiotomy, occiput posterior presentation (OP), epidural analgesia, spinal analgesia, use of nitrous oxide gas, paracervical block, maternal age, body mass index [BMI = body weight in kilograms/height in meters squared], length of active second stage of birth, birth weight, and head circumference. OP, spinal analgesia, BMI, length of active second stage of birth, and head circumference were adjusted for the years 2004-2007. All continuous variables (maternal age, BMI, birth weight, head circumference, and length of active 2^nd ^stage of birth) were classified as categorical variables.

Comparison of data for the teaching university hospitals and non-university hospitals showed that the OASIS rates of primiparous women were higher in teaching university hospitals (1.2% vs. 1.0%, p = 0.006), while there were non-significant differences between university and non-university hospitals in this respect for multiparous women (0.2% vs.0.2%). Adjustment for patient mix or the use of interventions did not significantly change the difference and the risk of primiparous women having OASIS was 13% (OR 0.87, 95% CI 0.80-0.96) lower in non-university hospitals, whereas for multiparous women hospital type did not significantly affect the risk of OASIS (OR 0.88, 95% CI 0.73-1.06) (data not shown).

## Discussion

The priority of the present study was to assess inter-hospital differences in the OASIS rate, patient mix and use of obstetric interventions. OASIS rates were compared over time, among university-based teaching hospitals and among non-university central hospitals. The availability of large national register data greatly facilitates studies such as this, especially for evaluating the occurrence and risk factors associated with rare complications such as OASIS. The most important limitations of register-based studies are in the reliability and coverage of the data. The present data were obtained from the mandatory, nationwide, population-based Medical Birth Register (MBR), which has been shown to provide excellent coverage and good data quality [6,15]. The data were checked at THL and returned for revision if necessary, and the check up was especially relevant to incidents that resulted in surgical repair with specific codes of diagnosis, requiring extra days of hospital care. In addition, the MBR covers all Finnish delivery hospitals and provides access to a vast amount of data. The information on OASISs was not available in the MBR before year 2004, but the data was taken from Hospital Discharge Register. Also this register is mandatory and its completeness and quality is high [16]. In 2006-2007, for example, it covered 95% of OASISs registered in the MBR.

The present study showed that the risk of OASIS depended on the hospital where the delivery occurred. Hospitals with high rates of OASIS for primiparous women also had high rate for multiparous women, implying that the quality of care might have played a crucial role in the observed variation. It may be speculated that midwifery care practices during the second stage of birth may also be an important factor in preventing OASIS. We found, for example, differences in the duration of active second stages, especially among university hospitals, presumably reflecting treatment differences. It is, however, of note that there might have been differences in definition of active second stage of birth. However, we were not able to investigate all important factors related to OASIS. For example, variations in techniques to protect the perineum used by midwives and pushing method have an influence on OASIS rates [17], but our register data did not contain that information. It is of note that there is no national protocol concerning obstetric management but the results of the present study challenged the current policy to optimize the process of giving birth. Further, previous studies have reported birth attendant's inexperience to be a risk factor for OASIS [18,19]. Making these practices visible within the medical records and routinely collected health registers would clarify the etiology of OASIS.

It might also be argued that the complication rates were under or over diagnosed or reported. Consequently, in the hospitals with higher rates of OASIS the recognition of the condition might have been better, but the lack of long-term sequelae, such as need for secondary repair or an increase in the incidence of anal incontinence, in the general population makes this assumption unlikely. Further, we suggest under reporting to be unlikely because the check up of the data was especially relevant to incidents that resulted in surgical repair with specific codes of diagnosis, requiring extra days of hospital care.

Generally, we found that previous inter-hospital comparisons were sparse and were aimed at assessing the extent of hospital variation in episiotomy use and its association with perineal outcomes, suggesting that lower episiotomy rates are associated with lower OASIS rates [20,21] which are not consistent with our results concerning obstetric care facilities where lateral episiotomy is exclusively used.

We believe that the results of the present study are likely to be generally applicable to hospitals in countries with very similar health care systems, providing free access to antenatal and obstetric services covering almost all deliveries. Furthermore, the risk profile of OASIS may be very different in countries with markedly lower or higher OASIS rates. The type of episiotomy used, and the very different roles of professionals on duty, may also considerably affect the outcomes.

## Competing interests

None declared. All authors declare, as researchers, independence from the funders and other conflicts of interest.

## Authors' contributions

All authors (SR, KV-J, MG, and SH) participated in designing the study. SR managed the dataset and performed statistical analyses. KV-J, MG, and SH gave advice regarding the statistical analyses. All authors contributed to the interpretation of the results, as well as to the writing and editing of the manuscript.
